# Bcl-2 expression related to altered p53 protein and its impact on the progression of human pancreatic carcinoma

**DOI:** 10.1038/sj.bjc.6690466

**Published:** 1999-06

**Authors:** Y-X Hu, H Watanabe, K Ohtsubo, Y Yamaguchi, A Ha, Y Motoo, T Okai, N Sawabu

**Affiliations:** Department of Internal Medicine and Medical Oncology, Cancer Research Institute, Kanazawa University, 4-86 Yoneizumi, Kanazawa 921-8044, Japan

**Keywords:** p53 protein, Bcl-2 protein, apoptosis, pancreatic carcinoma, immunohistochemistry

## Abstract

p53 and Bcl-2 are two important factors related to apoptosis and tumorigenesis. In this study, a series of 52 cases of pancreatic carcinoma (PC) were investigated using an immunohistochemical assay to determine whether altered expression of Bcl-2 and p53 has an impact on the progression of this malignancy. Cytoplasmic immunoreactivity for Bcl-2 and nuclear staining of p53 was found in 12 (23.1%) and 32 (63.5%) cases of PC respectively. Furthermore, an inverse correlation between the expression of p53 and Bcl-2 existed in this series (*P* < 0.01). In a subgroup, the proportion of tumours showing that p53-positive and Bcl-2-negative staining was increased with increasing histological grade and clinical stage (*P* < 0.05), and moreover, the survival period of those patients whose tumour had this staining was shorter than those with other staining patterns of combined p53 and Bcl-2 (*P* < 0.05). Therefore, it is concluded that simultaneously aberrant expression of Bcl-2 and p53 may confer PC with more malignant clinicopathological characteristics. © 1999 Cancer Research Campaign


*British Journal of Cancer* (1999) **80**(7), 1075–1079
© 1999 Cancer Research Campaign
Article no. bjoc.1998.0466

### Bcl-2 expression related to altered p53 protein and its impact on the progression of human pancreatic carcinoma

**Y-X Hu*, H Watanabe, K Ohtsubo, Y Yamaguchi, A Ha, Y Motoo, T Okai and N Sawabu**

Department of Internal Medicine and Medical Oncology, Cancer Research Institute, Kanazawa University, 4-86 Yoneizumi, Kanazawa 921-8044, Japan

**Summary** p53 and Bcl-2 are two important factors related to apoptosis and tumorigenesis. In this study, a series of 52 cases of pancreatic carcinoma (PC) were investigated using an immunohistochemical assay to determine whether altered expression of Bcl-2 and p53 has an impact on the progression of this malignancy. Cytoplasmic immunoreactivity for Bcl-2 and nuclear staining of p53 was found in 12 (23.1%) and 32 (63.5%) cases of PC respectively. Furthermore, an inverse correlation between the expression of p53 and Bcl-2 existed in this series ($P < 0.01$). In a subgroup, the proportion of tumours showing that p53-positive and Bcl-2-negative staining was increased with increasing histological grade and clinical stage ($P < 0.05$), and moreover, the survival period of those patients whose tumour had this staining was shorter than those with other staining patterns of combined p53 and Bcl-2 ($P < 0.05$). Therefore, it is concluded that simultaneously aberrant expression of Bcl-2 and p53 may confer PC with more malignant clinicopathological characteristics.

**Keywords:** p53 protein; Bcl-2 protein; apoptosis; pancreatic carcinoma; immunohistochemistry

A growing number of studies suggested that pancreatic carcinoma (PC) seems to be the result of a series of genetic alterations, including the oncogenes and tumour suppressor genes. The products of *p53* and *Bcl-2* genes are important factors associated with apoptosis and mechanisms underlying the malignant development and progression of some human tumours. The mutation of *p53* gene and overexpression of its product in PC, including cell lines and primary human resected tissues, have been extensively studied (Barton et al, 1991; Aizawa et al, 1996; Lundin et al, 1996; Ruggeri et al, 1997) but the molecular pathogenesis of Bcl-2 protein during the progression of this malignancy has not been well-characterized. In the only study published on this, Sinicrope et al (1996) reported Bcl-2 expression in 45.0% of PC. However, this result was obtained based on a limited number of samples including some ampullary adenocarcinomas. In the present study, we investigated Bcl-2 expression in a larger series to further evaluate the status of Bcl-2 expression, its correlation with the altered p53 protein, and impact on the progression of PC.

### MATERIALS AND METHODS

### Patients and tissues

Fifty-two patients with a final pathological diagnosis of primary pancreatic ductal adenocarcinoma and complete clinicopathological data were enrolled in this study. Cystadenocarcinoma and adenosquamous carcinoma of the pancreas were excluded. Histological grading and clinical staging were determined using criteria described in detail previously (Hermreck et al, 1974; Klöppel et al, 1985). Most tissues of PC were primary lesions ($n = 30$) and some were metastatic lesions ($n = 22$, including four liver tissues, and 18 regional or distant lymph nodes), all of which were obtained at Changhai Hospital, Second Military Medical University in Shanghai, China, and the Cancer Research Institute Hospital, Kanazawa University in Kanazawa, Japan. Twenty-eight patients had complete follow-up records. In addition, six normal tissues of the pancreas were included in this study as control.

*Received 16 April 1998*
*Revised 6 October 1998*
*Accepted 22 October 1998*

*Correspondence to:* N Sawabu

### IMMUNOHISTOCHEMICAL PROCEDURES

All specimens were fixed in 10% formalin and embedded in paraffin and cut into 4-μm-thick serial sections. A highly sensitive immunohistochemical staining was based on labelled streptavidin–biotin complex (SLAB; Dako, Carpinteria, CA, USA) and combined with the antigen retrieval method by microwave heating. In brief, after deparaffinization, the sections were treated for 20 min at 95°C using a microwave in 10 mM sodium citrate for Bcl-2 staining, and then incubated with blocking serum containing carrier protein and 15 mM sodium azide at room temperature for 20 min to block non-specific binding. Next, the sections were incubated at 4°C overnight with primary monoclonal antibody, anti-Bcl-2 (Clone 124; Dako, Glostrup, Denmark) diluted with 1/60, and then subjected to sequential 20-min incubation at room temperature with biotinylated link antibody peroxidase-labelled streptavidin. Staining was detected with AEC Substrate-Chromogen (Dako, Carpinteria, CA, USA) and the sections then counter-stained in haematoxylin and mounted using an aqueous medium. Negative control was performed by replacing primary antibody using phosphate-buffered saline (PBS). Lymphocytes in primary sites or tissues of metastatic PC served as an internal

*Y-X Hu is a postdoctoral fellow from Changhai Hospital, Second Military Medical University, Shanghai, China.

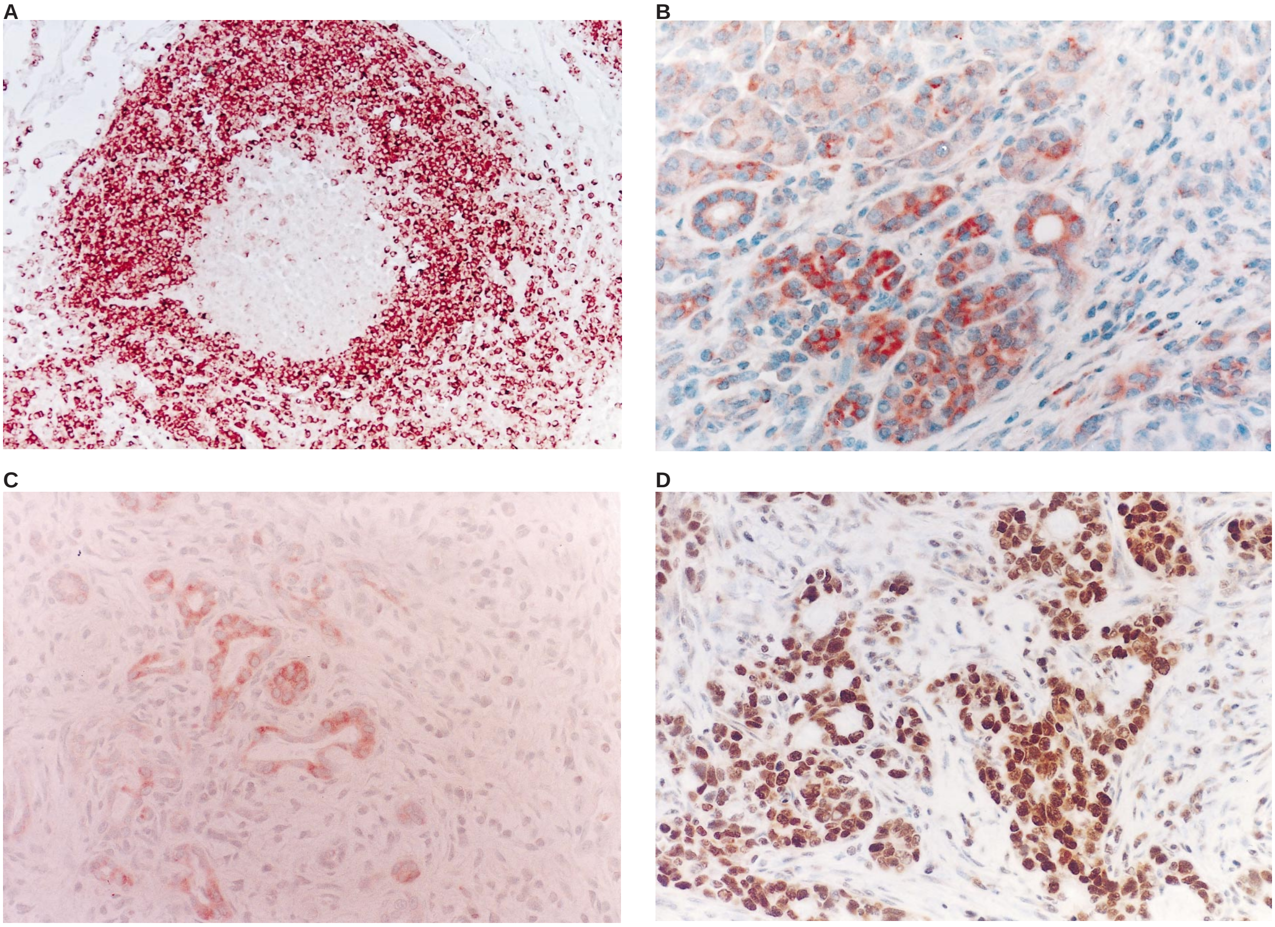

**Figure 1** (**A**) As an internal positive control, lymphocytes in metastatic sites of pancreatic adenocarcinoma are positive for Bcl-2-staining (× 200). (**B**) Expression of Bcl-2 in normal pancreas: the cytoplasm of acinar cells is partially stained (× 200). (**C**) Positive staining of Bcl-2 in a case of well-differentiated adenocarcinoma of the pancreas (× 200). Note Bcl-2 staining of lymphocytes in the stroma is also observed. (**D**) Strong nuclear staining of altered p53 protein is seen in almost all cancer cells (× 200)

**Table 1** Distribution of the four patterns of the expression of Bcl-2 and altered p53 proteins in PC

| Clinicopathological factors | No. of samples | Expression pattern | | | |
|---|---|---|---|---|---|
| | | p53+/Bcl-2– | p53+/Bcl-2+ | p53–/Bcl-2– | p53–/Bcl-2+ |
| Histological grade | | | | | |
| G1 | 15 | 4 (26.7) | 4 (26.7) | 4 (26.7) | 3 (20.0) |
| G2 | 21 | 10 (47.6) | 1 (4.8) | 9 (42.9) | 1 (4.8) |
| G3 | 16 | 11 (68.8) | 2 (12.5) | 2 (12.5) | 1 (6.3) |
| Clinical stage | | | | | |
| I | 4 | 1 (25.0) | 0 (0) | 2 (50.0) | 1 (25.0) |
| II | 16 | 6 (37.5) | 5 (31.3) | 3 (18.8) | 2 (12.5) |
| III | 15 | 8 (53.3) | 1 (6.7) | 5 (33.3) | 1 (6.7) |
| IV | 17 | 10 (58.8) | 1 (5.9) | 5 (29.4) | 1 (5.9) |

 © 1999 Cancer Research Campaign

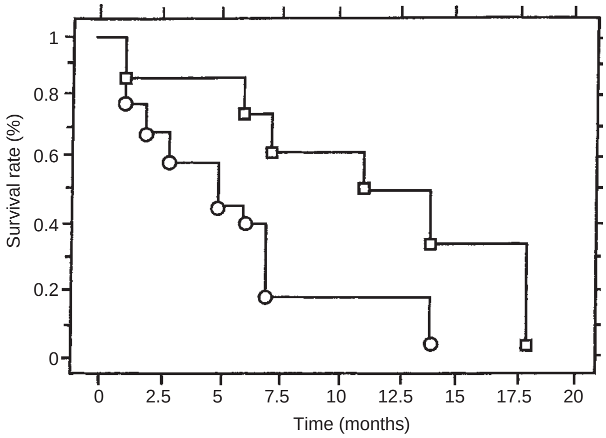

**Figure 2** Survival curve in patients with PC according to positive (□) and negative (○) staining for Bcl-2 protein

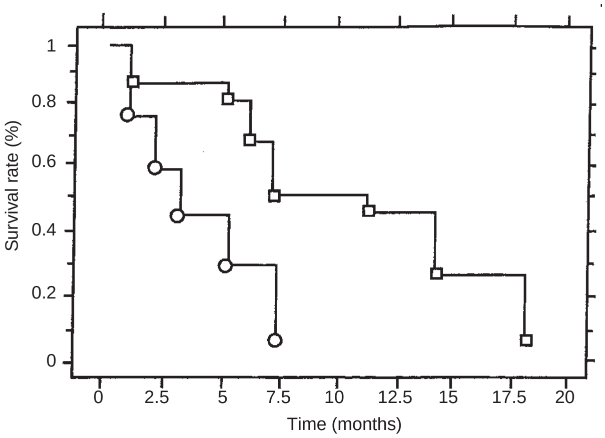

**Figure 3** Survival curve in patients with PC according to the expression of Bcl-2–/p53+ (○) and of Bcl-2–/p53+, Bcl-2+/p53+, Bcl-2–/p53–(□)

positive control for tumour tissues examined and external positive control for normal tissues of the pancreas without lymphocytes respectively. Staining procedures for the altered p53 protein were similar to those for Bcl-2 except that sodium citrate was replaced by Target Retrieval Solution (Dako, Carpinteria, CA, USA) in microwave treatment and diaminobenzidine (Sigma Chemical Co., St Louis, MO, USA) used as substrate for colouration. The primary antibody against the mutant p53 protein (DO7; Dako, Carpinteria, CA) was used at a dilution of 1:50. A case of colon adenocarcinoma, which was positive for p53 staining, was used as a positive control.

Staining results of Bcl-2 protein were judged according to the method established by Tron et al (1995) and the cytoplasmic staining of more than 5% of target cells was defined as positive. Nuclear staining of more than 25% of target cells was taken as the cut-off value for distinguishing positive from negative expression of altered p53 protein and the aim of this immunostaining classification was to indicate the presence of an underlying gene mutation (Cordon-Cardo et al, 1994).

### Statistical analyses

The $\chi^2$ test and McNemar's test were used to analyze the association between different variables. The survival analysis was determined according to the Kaplan–Meier method, and the statistical significance of the difference in survival distribution was evaluated by the log-rank test. A $P$-value of less than 0.05 was considered statistically significant.

### RESULTS

### Bcl-2 expression in PC

Positive immunoreactivity for Bcl-2 protein was detectable in the cytoplasm and also frequently on the nuclear membrane (Figure 1A). Bcl-2 expression was found in all six cases of normal pancreas, but the staining intensity was different among three kinds of cells of the pancreas: strong, in acinar cells (Figure 1B); moderate, in islet of Langerhans; and weak, in ductal epithelium. The staining pattern of Bcl-2 protein was variable among PC tissues. Some sections showed labelling of the vast majority of cells, whereas in others only a small area was focused to be positive for the Bcl-2 protein. In some of the positive sections of PC, focal and weak staining with strong staining in infiltrating lymphocyte was a striking feature. Twelve (23.1%) of 52 cases of PC expressed Bcl-2 protein (Figure 1C) and distributed in seven of 15 (46.7%) well-differentiated tumours (G1), two of 21 (9.5%) moderately differentiated (G2) and three of 16 (18.8%) poorly differentiated tumours (G3) as well as in 25.0% of clinical stage I, 68.9% of stage II, 13.3% of stage III and 11.8% of stage IV. Statistical analysis showed that positive staining for Bcl-2 was associated with tumour pathological grade (G1 vs G2 and G3; $P < 0.05$) and clinical stage (stage I and stage II vs stage III and IV; $P < 0.05$). Additionally, in 28 patients with follow-up records, eight cases had tumours that were positive and 20 cases that were negative for Bcl-2 staining. All but one (87.5%) Bcl-2-positive patients, but only nine in 20 (45.0%) patients with Bcl-2 negative staining, survived for 6 months or more after the operation, and a significant difference was observed between these two groups ($P < 0.05$, Figure 2).

### Expression of altered p53 protein in PC

Normal pancreas did not express the altered or mutant p53 protein. Unlike Bcl-2, p53-positive staining was more diffuse and intense. Thirty-two (61.5%) of PC showed nuclear p53-positive staining in more than 25% of tumour cells stained, among which 20 cases had strong and diffuse staining in more than 50% of cells (Figure 1D). Tumours with p53-positive staining distributed in 53.3% (8/15) of G1, 52.4% (11/21) of G2 and 81.3% (13/16) of G3, and positive rate of p53 staining in clinical stage I, II, III and IV was 25.0% (1/4), 68.8% (11/16), 60.0% (9/15) and 64.7% (11/17) respectively. However, p53 immunostaining did not correlate with tumour pathological grade, clinical stage and prognosis ($P > 0.05$).

### Relation of Bcl-2 expression to altered p53 in PC

Twenty-five (78.1%) of 32 tumours with p53-positive staining did not express Bcl-2. In addition, among 40 tumours with Bcl-2-negative staining, 25 cases (62.5%) expressed the altered p53 protein. There was a significantly inverse correlation between the expression of Bcl-2 and p53 ($P < 0.01$). Tumours examined, according to different combinations expressing these two proteins, were classified into the following four subgroups, i.e. p53+/Bcl-2– ($n$ = 25), p53+/Bcl-2+ ($n$ = 7), p53–/Bcl-2– ($n$ = 15) and

© 1999 Cancer Research Campaign p53–/Bcl-2+ ($n$ = 5). A trend toward rate increasing with increase of histological grade and clinical stage was found in the subgroup of p53+/Bcl-2– staining (Table 1). Additionally, among 28 patients with follow-up records, 12 demonstrated Bcl-2–/p53+ and 16 demonstrated three other patterns. Only three cases (25.0%) in the former subgroup, but ten of the other cases (62.5%), survived more than 6 months after the operation. Furthermore, statistical analysis showed that patients with p53+/Bcl-2– had a greater number of worse prognoses than those with the other three expression patterns of these two proteins ($P < 0.05$, Figure 3).

### DISCUSSION

In this study, we took into account the expression of Bcl-2 and its relation to the altered p53 protein in PC. Based on these results, we evaluated the impact of the altered expression of these two proteins on the progression of PC. Bcl-2 was expressed in normal pancreatic tissue examined, which is consistent with previous findings (Krajewski et al, 1994; Sinicrope et al, 1996). Interestingly, we also observed the presence of Bcl-2 expression in normal duodenal epithelial cells and hepatocytes adjacent to metastatic PC tissues. Actually, some studies have demonstrated that Bcl-2 expression in other human normal tissues is not uncommon (McDonnell et al, 1992; Yan et al, 1996).

The impact of Bcl-2 expression on the progression of PC has not been well-characterized. Sinicrope et al (1996) found that about 45.0% of PC expressed Bcl-2 protein, which is higher than that of our study. It is likely that case selection is responsible for this discrepancy, as all cases of PCs examined in the study of Sinicrope et al (1996) were resectable. The mutually exclusive expression of Bcl-2 and altered p53 protein shown in PC, in fact, also existed in the normal tissue of the pancreas. Our results showed that all normal tissues of the pancreas did not express the altered p53 but all cases expressed Bcl-2. Recently, a similarly inverse relation has been observed in other human cancers, including gastric lymphoma (Nakamura et al, 1996), lung cancer (Ishida et al, 1997) and breast carcinoma (Hurlimann et al, 1995). Our results and those described above, however, seem paradoxical given the known functions of Bcl-2 as an oncoprotein. Actually, the mechanisms regulating Bcl-2 expression appear to be different among human tissues and, furthermore, role of Bcl-2 could not fully be explained by its function of oncoprotein. The mutually exclusive expression of Bcl-2 and p53 shown in the present study raises a possibility that Bcl-2 protein could be down-regulated by the mutant protein. Similarly, Haldar et al (1994) reported that mutant p53 protein might down-regulate Bcl-2 in breast cancer cell, and Miyashita et al (1994) confirmed this using *Bcl-2/CAT* receptor gene plasmid and co-transfection assay. In a study of breast cancer done by Krajewski et al (1997), the percentage of Bcl-2-immunopositive tumour cells was found significantly lower in the p53-positive (median 20%) subset as compared to the p53-negative (median 85%) subsets. Concomitantly, we observed that altered p53 protein was expressed diffusely but Bcl-2 expression remained weak in some sections of PC. There have been some studies suggesting that the decreased expression of Bcl-2 confers more malignant biological behaviour and clinicopathological features on some types of tumour (Tron et al, 1995; Stattin et al, 1996; Ohbu et al, 1997; Tjalma et al, 1997). Furthermore, such role of Bcl-2 protein seems to be enhanced in the presence of the altered p53 protein. In the present study, we found that the absence of Bcl-2 expression was mainly observed in the population of highly malignant tumours (81% of G3 and 88% of clinical stage III and IV), moreover, most of which expressed altered p53 protein. Additionally, the finding of a strong association between simultaneously altered expression of these two proteins and poor prognosis in some patients with PC was consistent with the literature, suggesting that the expression of p53+/Bcl-2– has a bad impact on patient prognosis in at least some tumours (Pezzella et al, 1993; Haldar et al, 1994; Piris et al, 1994).

Bcl-2 has been considered to have function of blocking apoptosis or programmed cell death. However, the mechanisms underlying apoptosis are very complicated, some of which at the present are still unclear. Shiraki et al (1997) reported that liver metastasis of colon carcinoma was correlated with apoptosis, suggesting that programmed cell death might promote metastasis. We noticed that Bcl-2 antibody rarely stained tumour tissues in metastatic liver of PC, but normal hepatocytes and lymphocytes adjacent to cancer nest could be readily stained. Furthermore, among 17 tumour tissues with clinical stage IV in this series, all of which had liver or distant lymph node metastases, 15 (88.2%) cases did not express Bcl-2, suggesting that the absence of Bcl-2 expression was related to tumour metastasis. On the basis of these findings, it may be speculated that the altered expression of Bcl-2 and p53 during the development and progression of human PC is correlated to apoptosis to some extent, although the mechanism of apoptosis involved with the promotion of metastasis is unknown.

In accordance with the absence of Bcl-2 protein, the mutant p53 was reported to be involved in neovascularization or angiogenesis, tumour invasion and metastasis (Dameron et al, 1994; Kieser et al, 1994; Fontanini et al, 1997). Moreover, Yamanaka et al (1993) reported elevated messenger RNA and protein levels for acid fibroblast growth factor and basic fibroblast growth factor, two angiogenetic factors, in most PC tissues examined. Therefore, it is possible that the simultaneously altered expression involving Bcl-2 and p53 confers more malignant clinicopathological characteristics, including prognosis on some tumours (Piris et al, 1994; Hurlimann et al, 1995; Tjalma et al, 1997). On the other hand, we used not only primary tumours of PC but also metastases for analysis. Since it is known that metastases originate from a subgroup of cells, the expression of prognostic factors might be different in metastases than in the primary tumours. However, it seems possible that the specialized subpopulations of cells producing metastases pre-exist in heterogeneous cells of primary tumour, and they represent at least some characters of primary tumour.

Our study is only preliminary and neither of the explanations described above for simultaneously aberrant expression of Bcl-2 and p53 in PC is satisfying. Further correlative investigations on p53, Bcl-2 and another member of Bcl-2 family, especially on dynamic links among them, will provide useful information about the molecular complexity of genetic diagnosis or control of PC, one of the most malignant of all diseases.

### ACKNOWLEDGEMENTS

This work was supported in part by Grants-in-Aid for Scientific Research and Scientific Research on Priority Areas from the Japanese Ministry of Education, Science, Sports, and Culture; and by a grant from Mitsui Life Social Welfare Foundation.

### REFERENCES

Aizawa S, Sasaki M, Wada RI, Koyama M and Yagihashi S (1996) p53 protein expression in pancreatic tumors and its relationship to clinicopathological factors and prognosis. *J Surg Oncol* **62**: 279–283

© 1999 Cancer Research Campaign

Barton CM, Staddon SL, Hughes CM, Hall PA, O'Sullivan C and Klöppel G (1991) Abnormalilities of the p53 tumor suppressor gene in human pancreatic cancer. *Br J Cancer* **64**: 1076–1082

Cordon-Cardo C, Dalbagni D, Saez G, Oliva MR, Zhang ZF, Rosai J, Reuter VE and Pellicer A (1994) TB53 muations in human bladder cancer: genotypic versus phenotypic patterns. *Int J Cancer* **56**: 347–353

Dameron KM, Volpert OG, Tainsky MA and Buck N (1994) Control of angiogenesis in fibroblast by p53 regulation of thrombospondin. *Science* **265**: 1582–1584

Fontanini G, Vignati S, Lucchi M, Mussi A, Calcinai A, Boldrimi L, Chine S, Silverstri V, Angelletti CA, Basolo F and Bevilacqua G (1997) Neoangiogenesis and p53 protein in lung cancer: their prognostic role and their relation with vascular endothelial growth factor (VEGF) expression. *Br J Cancer* **75**: 1295–1301

Haldar S, Negrini M, Monne M, Sabbioni S and Croce CM (1994) Down-regulation of Bcl-2 by p53 in breast cancer cells. *Cancer Res* **54**: 2095–2057

Hermreck AS, Thomas COY and Friezes SR (1974) Importance of pathologic staging in the surgical management of adenocarcinoma of the exocrine pancreas. *Am J Surg* **85**: 653–657

Hurlimann J, Larrinaga B and Vala DL (1995) *bcl-2* protein in invasive ductal breast carcinomas. *Virchows Arch* **426**: 163–168

Ishida H, Irie K, Itoh T, Furkukawa T and Tokunaga O (1997) The prognostic significance of p53 and *bcl-2* expression in lung adenocarcinoma and its correlation with Ki-67 growth fraction. *Cancer* **80**: 1034–1045

Kieser A, Weich HA, Brandner G, Marmé D and Kolch W (1994) Mutant p53 potentiates protein kinase C introduction of vascular endothelial growth factor expression. *Oncogene* **9**: 964–969

Klöppel G, Lingenthal G, Vonbülow M and Kern HF (1985) Histological and fine structural features of pancreatic ductal adenocarcinoma in relation to growth and prognosis: studies in xenografted tumors and clinicopathological correlation in a series of 75 cases. *Histopathology* **9**: 841–856

Krajewski S, Krajewska M, Shabaik A, Miyashita T, Wang H-G and Reed JC (1994) Immunohistochemical determination of in vivo distribution of Bax, a dominant inhibitor of Bcl-2. *Am J Pathol* **145**: 1323–1333

Krajewski S, Thor AD, Edgerton SM, Moore DH, Krajewska M and Reed JC (1997) Analysis of Bax and Bcl-2 expression in p53-immunopositive breast cancers. *Clin Cancer Res* **3**: 199–208

Lundin J, Nordling S, von Boguslawsky K, Roberts PJ and Haglund C (1996) Prognostic value of immunohistochemical expression of p53 in patients with pancreatic cancer. *Oncology* **53**: 104–111

McDonnell TJ, Troncoso P, Brisbay SM, Logothetis C, Chung LW, Hsiek JT, Tu SM and Campbell ML (1992) Expression of prooncogene Bcl-2 in the prostate and its associated with emergence of androgen-independent prostate cancer. *Cancer* **52**: 6940–6947

Miyashita T, Harigai M, Hanada M and Reed JC (1994) Identification of a p53-dependent negative response element in the *bcl-2* gene. *Cancer Res* **54**: 3131–3135

Nakamura S, Akazawa N, Yao T and Tsuneyoshi M (1996) Inverse correlation between the expression of *bcl-2* and p53 protein in primary gastric lymphoma. *Hum Pathol* **27**: 225–233

Ohbu M, Saegusa M, Kobayashi N, Tsukamoto H, Mieno H, Kakita A and Okayasu I (1997) Expression of *bcl-2* protein in esophageal squamous cell carcinomas and its association with lymph node metastasis. *Cancer* **79**: 1287–1293

Pezzella F, Turley H, Kuzu I, Tungekar FM, Dunnil SM and Pierce CB (1993) bcl-2 protein in non-small-cell lung carcinoma. *N Engl J Med* **329**: 690–694

Piris MA, Pezzella F, Martinez-Montero JC, Orradre JL, Villuendas R, Sanchez-Beato M, Cuena R, Cruz MA and Martinez B (1994) p53 and bcl-2 expression in high-grade B-cell lymphomas: correlation with survival time. *Br J Cancer* **69**: 337–341

Ruggeri BA, Huang L, Berger D, Chang H, Klein-Szanto AJP, Goodrow T, Wood M, Obara Y, Heath CW and Lynch H (1997) Molecular pathology of primary and metastatic ductal pancreatic lesions: analyses of mutations and expression of the p53, mdm-2, and p21/WAF-1 genes in sporadic and familial lesions. *Cancer* **79**: 700–716

Shiraki K, Tsuji N, Shioda T, Isselbacher KJ and Takahashi H (1997) Expression of Fas ligand in liver metastases of human colonic adenocarcinoma. *Proc Natl Acad Sci USA* **94**: 6420–6425

Sinicrope FA, Evans DB, Leach SD, Cleary KR, Fenoglio CJ, Lee JJ and Abbruzzese JL (1996) bcl-2 and p53 expression in respectable pancreatic adenocarcinoma: associated with clinical outcome. *Clin Cancer Res* **2**: 2015–2022

Stattin P, Damber JE, Karlberg L, Nordgren H and Bergh A (1996) Bcl-2 immuno-reactivity in prostate tumorigenesis in relation to prostatic intraepithelial neoplasia, grade, hormonal status, metastatic growth and survival. *Urol Res* **24**: 257–264

Tjalma W, Weyler J, Goovaerts G, De-Pooter C, Van-Marck E and van-Dam P (1997) Prognostic value of bcl-2 expression in patients with operable carcinoma of the uterine cervix. *J Clin Pathol* **50**: 33–36

Tron VA, Krajewski S, Klein-Parker H, Li G and Reed JC (1995) Immunohistochemical analysis of Bcl-2 protein regulation in cutaneous melanoma. *Am J Pathol* **146**: 643–650

Yamanaka Y, Friess H, Buvhler M, Beger HG, Uchida E, Onda M, Kobin MS and Konc M (1993) Overexpression of acidic and basic fibroblast growth factors in human pancreatic cancer correlates with advanced tumor stage. *Cancer Res* **53**: 5289–5296

Yan J-J, Chen F-F and Jin Y-T (1996) Immmunohistochemical detection of Bcl-2 protein in small cell lung carcinomas. *Oncology* **53**: 6–11

*© 1999 Cancer Research Campaign*
